# Influence of tebuconazole and copper hydroxide on phosphatase and urease activities in red sandy loam and black clay soils

**DOI:** 10.1007/s13205-016-0367-0

**Published:** 2016-02-23

**Authors:** B. Anuradha, A. Rekhapadmini, V. Rangaswamy

**Affiliations:** Department of Microbiology, Sri Krishnadevaraya University, Anantapuramu, Andhra Pradesh 515 003 India

**Keywords:** Copper hydroxide, Groundnut soils, Phosphatase, Tebuconazole, Urease

## Abstract

The efficacy of two selected fungicides i.e., tebuconazole and coppoer hydroxide, was conducted experiments in laboratory and copper hydroxide on the two specific enzymes phosphatase and urease were determined in two different soil samples (red sandy loam and black clay soils) of groundnut (*Arachis hypogaea* L.) from cultivated fields of Anantapuramu District, Andhra Pradesh. The activities of the selected soil enzymes were determined by incubating the selected fungicides-treated (1.0, 2.5, 5.0, 7.5 and 10.0 kg ha^−1^) and -untreated groundnut soil samples at 10 day intervals. By determining the effective concentration, the rate of selected enzyme activity was estimated by adding the suitable substrate at 10, 20, 30 and 40 days of soil incubation. Both the enzyme activities were increased up to 5.0 kg ha^−1^ level of fungicide in both soil samples significantly at 10 days of soil incubation and further enhanced up to 20 days of incubation. The activity of the phosphatase and urease decreased progressively at 30 and 40 days of incubation. From overall studies, higher concentrations (7.5 and 10.0 kg ha^−1^) of both tebuconazole and copper hydroxide were toxic to phosphatase and urease activities, respectively, in both soil samples.

## Introduction

In India, it has become a common trend in modern agriculture to apply agrochemicals such as pesticides to increase agricultural productivity and has become an integral part of agriculture. About one-third of the world’s food crop is destroyed by pests annually (Punitha et al. 2012). Different groups of pesticides with various formulations have been introduced into agricultural soils either simultaneously or in succession to protect crops from weeds, insects, and fungi. Pesticides have beneficial impacts in improving and stabilizing agricultural productivity by controlling pests; however, these organic chemicals may contaminate the soil ecosystem and various groups of organisms in soil, which play a vital role in mineralization, nitrification and phosphorus recycling. Among the different groups of organisms, microorganisms play an important role in many soil biological processes; soil enzymes play various biochemical functions in the overall process of organic matter decomposition in the soil system (Burns 1983; Sinsabaugh et al. 1991). The soil activities undergo complex biochemical processes that consist of integrated and ecologically connected synthetic processes, immobilization and enzyme stability (Khaziyev and Gulke 1991). Many investigations were performed to study the effect of various pesticides on different enzyme activities in soils of different origin (Sannino and Gianfreda 2001). The application of various pesticides may adversely affect not only the soil microflora but also soil enzymes and physico-chemical properties such as pH, salinity and alkalinity that cause damage to the fertility of the soil. The enzymatic activities in soil are mainly from microbial origin, and are derived from intracellular, cell associated or free enzymes (Mohiuddin and Mohammed 2014). Therefore, it is clear that there is a close relationship between pesticides and microorganisms, because these chemicals may have a deleterious effect on non-target microorganisms and most of these pesticides could be metabolized by different microorganisms resulting in the modification of their activity (Walia et al. 2014).

India, Anantapuramu, a semiarid region of Andhra Pradesh, ranks first in the area of groundnut (*Arachis hypogaea* L.) cultivation, but its productivity is low and fluctuates around 9 q ha^−1^ on average (Anonymous 2014). Groundnut is one of the important major profitable oil seed crop grown throughout the year in India (Guha and Chandrasekhar 2001) and contributes to 41.3 % of the country’s oilseed production (Giraddi et al. 1999). The monocropping of groundnut with high-yielding varieties resulted in an eruption of fungi, causing damage to groundnut crop in many areas of Andhra Pradesh; more than 90 insect pests and mites were found to be associated with the groundnut crop (Das et al. 1995). To protest the groundnut crop loss and to get higher yield, the chemical use of pesticides has become an essential part in modern agriculture (Parama et al. 1997). Reports were available on the influence of pesticides on microbial activity with a different methodological approach (Gianfreda et al. 1994, 1995; Sannino and Gianfreda 2001).

In the present work, the influence of two fungicides, tebuconazole and copper hydroxide, on the activities of phosphatase and urease enzymes of selected soil samples were investigated. These two enzymes have been selected because they play an important role in the carbon–nitrogen and phosphorus–sulfur cycles in soils. Phosphatase, which is an exracellular enzyme produced by many soil microorganisms, is responsible for the hydrolysis of organic phosphorus compounds to inorganic phosphorus (Monkiedje and Spiteller 2002). Phosphatase carries out a broad range of intracellular as well as soil-accumulated activities that catalyze the hydrolysis of both the esters and anhydrides of phosphoric acid. Urease in general helps to maintain nitrogen in the form of ammonia (NH_4_^+^) and is less leachable (Srinivasulu and Rangaswamy 2014). Urease catalyzes the hydrolysis of urea to CO_2_ and NH_4_^+^ ions by acting on C–N non-peptide bonds in linear amides (Ramudu et al. 2012). Urease in particular is a useful indicator to evaluate soil pollution. The application of pesticides to soil decreases the urease activity that reduces the hydrolysis of urea which is beneficial to soil fertility (Anuradha et al. 2015).

Many synthetic fungicides were developed in the 1940–1950s based on ‘old fungicide chemistry’ (Wightwick et al. 2013). These fungicides have a broad-spectrum multisite mode of action and are applied at high concentration rates. Many other fungicides were developed since 1970 based on ‘modern fungicide chemistry’, which have specific modes of action, and greater activity was applied at relatively lower application rates (Russell 2005). Few laboratory studies have been reported on the impact of fungicides on soil enzyme activity with contradictory effects. In the present study, a triazole fungicide tebuconazole and copper-based fungicide copper hydroxide were used based on their efficacy on soil enzymes. Tebuconazole, which is a systemic fungicide with protective action, is used as a spray that controls the leaf spot of groundnut. It is a potent xenobiotic to which exposure can cause metabolic alterations and lead to death of different organisms (Sehnem et al. 2010). The IUPAC name of tebuconazole is [(RS)-1-*p*-chlorophenyl-4,-dimethyl-3-(1H-1,2,4-triazol-1-ylmethyl) pentan-3-ol] (Potter et al. 2005). Copper-based fungicides that cause reduction in the populations of fungi and actinomycetes and inhibit nitrification, as well as to stimulate bacterial populations have been reported (Hussain et al. 2009; Lo 2010). In addition, copper hydroxide was selected as it represents a traditional Cu-based fungicide that has been in use for over 50 years in many parts of the world.

With this view, a laboratory study was conducted on the effect of tebuconazole and copper hydroxide on phosphatase and urease enzyme activities in selected soil samples. A little information is available on the behavior of tebuconazole and copper hydroxide on soil enzyme activities. The objective of the present study was to evaluate the response of the selected fungicides on phosphatase and urease enzyme activities. Hence in the present laboratory experiment, attempts were made to update the effect of selected fungicides on phosphatase and urease enzyme activities in groundnut soils, the major crop grown in Anantapuramu District, Andhra Pradesh, India.

## Materials and methods

### Soils used in the present study

Two soil samples, a black clay soil and red sandy loam soil, were collected from groundnut-cultivated fields of Anantapuramu District, Andhra Pradesh, India. Soil samples with a known history of pesticide use were chosen from a depth of 12 cm and mixed thoroughly to prepare a homogenous composite sample, air-dried at room temperature and sieved through a 2 mm sieve and stored at 4 °C.

### Analysis of the physico-chemical characteristics of soil samples

Mineral matter of soil samples such as sand, silt and clay contents were analyzed with the use of different sizes of sieves by following the method of Alexander (1961). Water-holding capacity (WHC) of the soil samples was determined by adding distilled water up to the saturation point and then 60 % water-holding capacity of the soil samples was calculated by Johnson and Ulrich (1960). The pH of the soil samples was determined by mixing soil and water in the ratio of 1:1.25 using Systonics digital pH meter with calomel glass electrode assembly. Organic carbon content in soil samples was estimated by Walkey–Black method and the organic matter was calculated by multiplying the values with 1.72 (Jackson 1971). The electrical conductivity of soil samples after addition of 100 ml distilled water to 1 g soil samples was measured by a conductivity bridge. The total nitrogen content in soil samples was determined by the method reported by Jackson (1971). The inorganic ammonium-nitrogen content in soil samples after extraction of 1 M KCl by the Nesslerization method Jackson (1971) and the contents of nitrite-nitrogen were determined by the method reported by Barnes and Folkard (1951), and the contents of nitrate-nitrogen by Brucine method Ranney and Bartlett (1972) after extraction with distilled water were determined. The important physico-chemical characters of the two soil samples are listed in Table 1.

**Table 1:** Physico-chemical properties of soils used in the present study

Properties	Black clay soil	Red sandy loam soil
Sand (%)	72.6	53.4
Silt (%)	18.3	27.8
Clay (%)	9.1	18.8
pH^a^	7.8	6.5
Water-holding capacity (ml g^−1^ soil)	0.43	0.23
Electric conductivity (m mhos)	264	228
Organic matter^b^ (%)	1.206	0.67
Total nitrogen^c^ (%)	0.078	0.045
NH_4_^+^-N (µg g^−1^ soil)^d^	7.09	6.01
NO_2_^−^-N (µg g^−1^ soil)^e^	0.62	0.48
NO_3_^−^-N (µg g^−1^ soil)^f^	0.98	0.81

^a^1:1.25 (soil:water)

^b^Walkley–Black method (Jackson 1971)

^c^Micro-Kjeldhal method (Jackson 1971)

^d^Nesslerization method (Jackson 1971)

^e^Diazotization method (Barnes and Folkard 1951)

^f^Brucine method (Ranney and Bartlett 1972)

### Fungicides used in the in the present study

Fungicides, tebuconazole (25.9 % m/m EC a soluble liquid) obtained from Makheshim-Agan, India, and copper hydroxide (77 % WP soluble powder) obtained from Bayer’s Science, India, of commercial grades were used for experimental purpose.

## Enzymes used in the present study

### Phosphatase activity (E.C. 3.1.1.1)

The activity of phosphatase under the influence of the fungicides at different concentrations was determined in black clay and red sandy loam soils. Two gram portions of soil samples were transferred into test tubes (12 × 125 mm) were treated with two fungicides to provide final concentrations of 10, 25, 50, 75 and 100 µg g^−1^ soil (equivalent to 1.0, 2.5, 5.0, 7.5 and 10.0 kg ha^−1^ field application rates). The soil samples without fungicide treatment served as control. All the treatments including controls were incubated in the laboratory at 28 ± 4 °C by maintaining 60 % water-holding capacity. After 10 days of incubation period, triplicate soil samples were withdrawn for the assay of phosphatase (Tabatabai and Bremner 1969; Srinivasulu et al. 2012). Similarly, the influence of the two insecticides at stimulatory concentration (5.0 kg ha^−1^) on the rate of phosphatase activity in two different soils was also determined in triplicate soil samples at 10, 20, 30 and 40 days of incubation.

### Assay of phosphatase

For the assay of phosphatase activity, each soil sample was treated with 6 ml of 0.1 M maleate buffer (pH 6.5) and 2 ml of 0.03 M *p*-nitrophenyl phosphate and the tubes incubated at 37 °C for 30 min. After incubation, the tubes were placed on ice before the soil extracts were passed through Whatman No.1 filter paper. To suitable aliquots of the extract, 1 ml of 5 M CaCl_2_ and 4 ml of 0.05 M NaOH were added and the yellow color developed was read at 405 nm in a spectrophotometer (Milton Roy Company).

### Urease activity (EC. 3.5.1.5)

For estimating the enzymatic activity of urease, 1 g portions of soil samples (black clay and red sandy loam soils) placed in 15 × 150 mm test tubes were treated with 1 ml of aqueous solutions of two fungicides to provide different concentrations of 1.0, 2.5, 5.0, 7.5 and 10.0 kg ha^−1^. The soil samples without insecticide treatment served as control. All the treatments including control were maintained at 60 % WHC and the tubes were incubated at 28 ± 4 °C. After 10 days of incubation, triplicate soil samples were withdrawn for the assay of urease.

### Assay of urease

Fawcett and Scott (1960) described the assay of urease at the desired intervals; 1 ml of 3 % urea and 2 ml of 0.1 M phosphate buffer (pH 7.1) were added to 1 g soil and the tubes were incubated at 37 °C for 30 min in a water bath. After incubation, the tubes were shaked and placed in ice until the ammonia was extracted with 10 ml of 2 M KCl. 5 ml of phenol–sodium nitroprusside solution and 3 ml of 0.02 M sodium hypochlorite were added to 4 ml of the filtrate. The mixture was shaken, incubated for 30 min in dark and the developed blue color was measured at 630 nm in a Spectronic 20 D spectrophotometer. After determining the effective concentration, the experiment was carried out further for 20, 30 and 40 days and assayed similarly.

### Statistical analysis

The concentrations of the phosphatase and urease enzymes were calculated on soil weight (over dried) basis. The insecticide treatments with untreated controls and the significant levels *p* ≤ 0.05 between the values of each sampling for each fungicide were obtained using SYSTAT statistical software package to find the results of Duncan’s multiple range (DMR) test (Megharaj et al.^1999^).

## Results and discussion

From the laboratory experiments, it was observed that there exists a relationship between soil enzymatic activities and the selected fungicide concentrations in the selected soil samples. On hydrolysis, the substrate *p*-nitrophenyl disodium orthophosphate was added, phosphatase was increased in both the selected soil samples with the selected fungicides tebuconazole and copper hydroxide than in the control at 1.0, 2.5 and 5.0 kg ha^−1^ levels, incubated for 20 days. Our investigation revealed that phosphatase and urease activities were drastically decreased at higher concentrations (7.5 and 10.0 kg ha^−1^) of tebuconazole and copper hydroxide-treated soils than the untreated controls throughout the experiment. The data obtained from these experiments are represented in Tables 2 and 3. The maximum enhancement in phosphatase activity over control was noticed in the black clay soil and red sandy loam soils at 20 days of incubation period. Further incubation periods, i.e., 30 and 40 days, the activity was decreased slowly in both the soil samples (Table 4).

**Table 2:** Influence of selected fungicides on activity of phosphatase in black clay soil after 10 days

Fungicide concentration (kg ha^−1^)	Tebuconazole	Coppe hydroxide
0.0	280 ± 4.52f (100)	280 ± 4.52f (100)
1.0	370 ± 3.48d (132)	420 ± 3.62d (142)
2.5	620 ± 2.94c (221)	610 ± 2.97c (217)
5.0	900 ± 0.87a (321)	870 ± 0.91a (310)
7.5	730 ± 2.16b (260)	710 ± 2.42b (253)
10.0	300 ± .22e (107)	430 ± 0.68e (153)

µg of *p*-nitrophenol (PNP) g^−1^ soil formed after 24 h incubation with *p*-nitro phenyl phosphate (PNPP)

Figures in parentheses indicate relative production percentages

Means, in each column, followed by the same letter are not significantly different (*P* ≤ 0.05) from each other according to Duncan’s multiple range (DMR) test

**Table 3:** Influence of selected fungicides on the activity of phosphatase in red sandy loam soil after 10 days

Fungicide concentration (kg ha^−1^)	Tebuconazole	Copper hydroxide
0.0	190 ± 3.14f (100)	190 ± 1.35f (100)
1.0	310 ± 2.42d (163)	350 ± 1.28d (183)
2.5	480 ± 3.62b (252)	490 ± 3.46c (257)
5.0	890 ± 2.47a (468)	840 ± 0.84a (442)
7.5	670 ± 1.86c (352)	610 ± 0.48b (321)
10.0	240 ± 1.64e (126)	290 ± 0.96e (152)

µg of *p*-nitrophenol (PNP) g^−1^ soil formed after 24 h incubation with *p*-nitrophenyl phosphate (PNPP)

Figures in parentheses indicate relative production percentages

Means in each column followed by the same letter are not significantly different (*P* ≤ 0.05) from each other according to Duncan’s multiple range (DMR) test

**Table 4:** Influence of selected fungicides on urease activity in black clay soil after 10 days

Fungicide concentration (kg ha^−1^)	Tebuconazole	Copper hydroxide
0.0	260 ± 2.47f (100)	260 ± 1.85f (100)
1.0	410 ± 0.68d (157)	420 ± 2.68e (161)
2.5	750 ± 2.81c (288)	650 ± 2.31c (250)
5.0	920 ± 2.46a (353)	800 ± 0.82a (307)
7.5	820 ± 2.46b (315)	740 ± 0.97b (284)
10.0	380 ± 1.48e (146)	350 ± 0.62d (134)

µg ammonia g^−1^ soil formed after 30 min incubation at 37 °C with urea

Figures, in parentheses, indicate relative production percentages

Means in each column followed by the same letter are not significantly different (*P* ≤ 0.05) from each other according to Duncan’s multiple range (DMR) test

Urease activity, implicated in the hydrolysis of urea, was significantly enhanced by the tebuconazole and copper hydroxide selected up to 5.0 kg ha^−1^ in both the soil samples when compared with the controls. However, the higher concentrations (7.5 and 10.0 kg ha^−1^) were toxic to the urease activity after 20 days of incubation period. The amount of ammonia formed from urea was more in soil samples treated with 2.5 and 5.0 kg ha^−1^ of tebuconazole, and copper hydroxide was maximum in black soil with tebuconazole and copper hydroxide at 5.0 kg ha^−1^. In the present experiment, higher levels of 7.5 and 10.0 kg ha^−1^ of the selected fungicides inhibited urease activity in two soils after 30 and 40 days of incubation periods (Table 5).

**Table 5:** Influence of selected fungicides on urease activity in red sandy loam soil after 10 days

Fungicide concentration (kg ha^−1^)	Tebuconazole	Copper hydroxide
0.0	300 ± 1.94f (100)	300 ± 2.42f (100)
1.0	490 ± 1.72d (163)	430 ± 2.62d (143)
2.5	580 ± 1.29c (193)	570 ± 0.85c (190)
5.0	910 ± 1.93a (303)	770 ± 2.48a (256)
7.5	690 ± 2.56b (230)	680 ± 1.91b (226)
10.0	420 ± 3.62e (140)	470 ± 0.74e (156)

µg ammonia g^−1^ soil formed after 30 min incubation at 37 °C with urea

Figures in parentheses indicate relative production percentages

Means, in each column, followed by the same letter are not significantly different (*P* ≤ 0.05) from each other according to Duncan’s multiple range (DMR) test

### Phosphatase activity

**Fig. 1:**
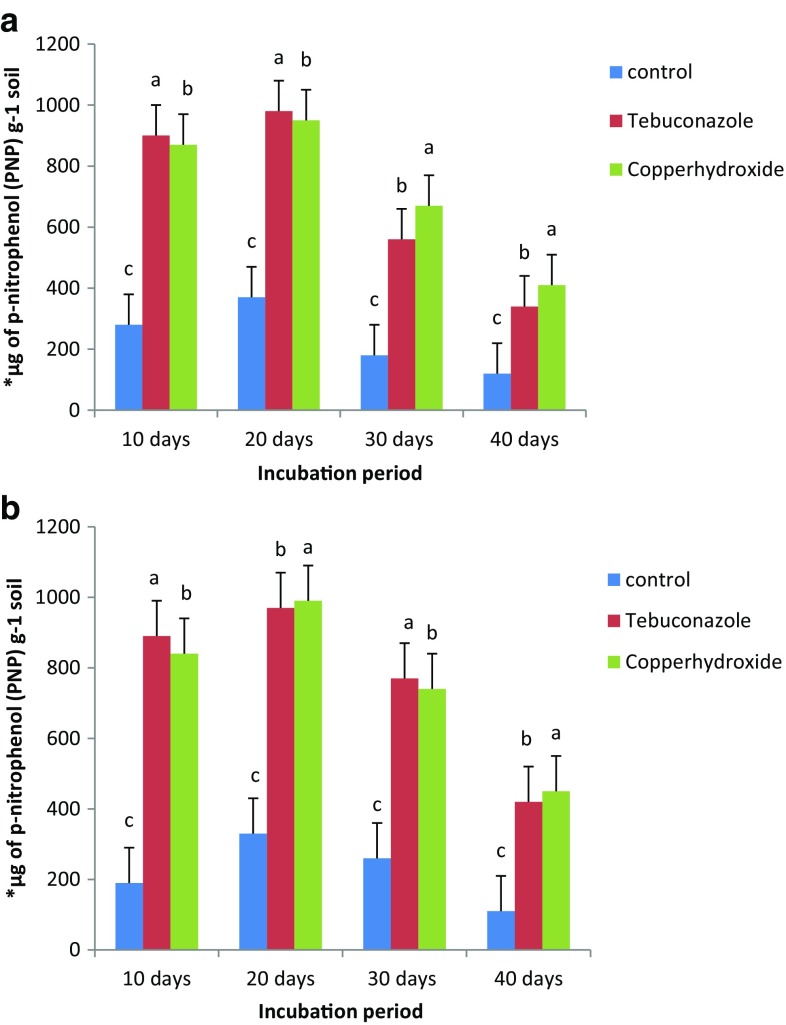
**a**, **b** Influence of tebuconazole and copper hydroxide on phosphatase^a^ activity in black clay and red sandy loam soil at 5.0 kg ha^−1^. *Asterisk* µg of *p*-nitrophenol (PNP) g^−1^ soil formed after 24 h of incubation with *p*-nitrophenyl phosphate (PNPP), after 10, 20, 30 and 40 days of incubation. The values are the mean ± SE for each incubation period and are not significantly different (*P* ≤ 0.05) from each other according to Duncan’s multiple range (DMR) test

From the results obtained from the present study, about 32–221 and 42–210 % increase in phosphatase activity over the control was noticed in the black soil with 20 days of incubation, whereas in the case of red sandy loam soil, the corresponding figures of the percentage enhancement by the two selected fungicides at two levels were 63–368 and 83–342 % during the same period of incubation. In comparison, tebuconazole at 5.0 kg ha^−1^ produced maximum stimulation in phosphatase activity in black clay soil than red sandy loam soil. At higher concentrations, i.e., 7.5 and 10.0 kg ha^−1^, phosphatase activity was significantly inhibited by treating the selected soil samples with both the selected fungicides. Among the two fungicide treatments, tebuconazole produced a different stimulation over the control. In the present study, comparatively, black soil showed higher enzyme activity than red soil throughout the experiment. Phosphatase activity was significantly inhibited at higher concentrations, i.e., 7.5 and 10.0 kg ha^−1^ in both the fungicide treatments, gradually with the incubation periods, i.e., 20, 30 and 40 days (Rangaswamy and Venkateswarlu 1996). Therefore in the present experiment, the maximum inhibition was recorded after 30 and 40 days of incubation periods. The enhancement in phosphatase activity over control was noticed in the black soil after 20 days of incubation. In the case of red sandy loam soil, the enhancement by the selected fungicides was also obtained at 5.0 kg ha^−1^ for 20 days of incubation and recorded in Table 2. The inhibitory effect was reduced upon further incubation, i.e., 30 and 40 days, due to the reduction in the concentration and degradation of the applied fungicides (Fig. 1).

Gianfreda et al. (1994, 1995) and Tabatabai and Bremner (1969) described the enzymatic systems which stimulate free enzymes in the soil solution and enzyme–soil colloid associations were considered. The effects of some selected pesticides on the activity of these enzymatic systems were studied. The results clearly indicate that the initial days of application of fungicides drastically reduced the number of bacteria and beneficial microflora, i.e., phosphate solubilizing bacteria in fungicide-amended soils when compared with the control soil. Unlike the previous study, there was some reported inhibition of phosphatase activity by captan (Chen and Edwards 2001; Piotrowska-Seget et al. 2008). Walia et al. (2014) reported the results on phosphatase activity with mancozeb, indicating the maximum average phosphatase activity at zero ppm (121.8 U) and the average activity decreased significantly to 113.2 U at 100 ppm mancozeb concentration. As the incubation period increases, there was decreased average phosphatase activity with no significant difference between various tebuconazole and copper hydroxide concentrations used in the present study. The decrease in the enzyme activity may be due to the decrease in the microbial population, destruction or inactivation of preexisting soil enzymes and the substrate limiting for enzyme induction. From the present study, the process of phosphorus solubilization was not distributed by the selected fungicide treatment. Ahemad and Khan (2011) showed that P-solubilization was in a minor way affected at recommended doses, but majorly affected at higher doses of tebuconazole and copper hydroxide. This may be because the soil microbial population can solubilize the added insoluble phosphates and is enriched in the presence of higher concentrations of the selected fungicides. In addition to that, the total population of microorganisms was decreased and resulted in the lesser utilization of the released phosphorus. For instance, tebuconazole is a potent xenobiotic, the exposure to which can cause metabolic alterations and the death of different microorganisms (Sehnem et al.  2010). Wightwick et al. (2013) reported that the interactive effects of Cu on the toxicity of captan and trifloxystrobin to soil microbial functions are not known and the toxicity of the alternative synthetic organic fungicide compounds may be increased if they are additive effects with Cu. Exposure to prolonged Cu may result in the alteration of the susceptibility of soil microbial population to the additional stress caused by application of these synthetic fungicide compounds.

### Urease activity

**Fig. 2:**
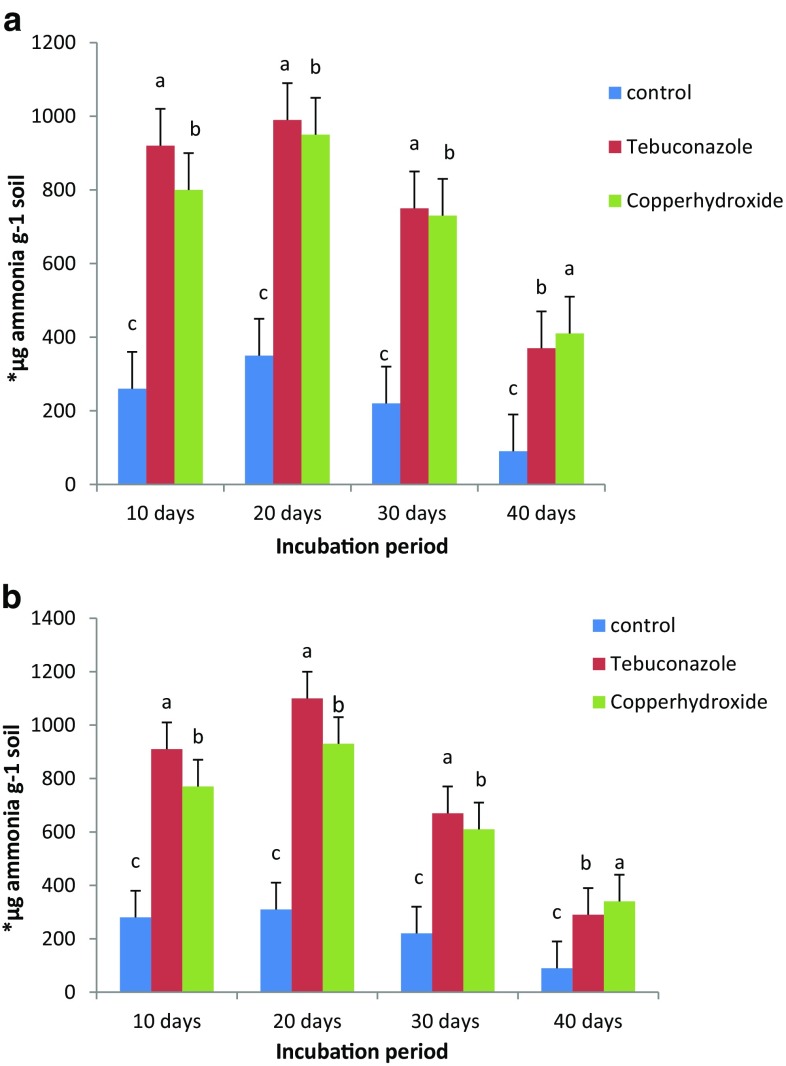
**a, b** Influence of tebuconazole and copper hydroxide on urease (*asterisk*) activity in black clay and red sandy loam soil at 5.0 kg ha. *Asterisk* µg ammonia g^−1^ soil formed after 30 min incubation at 37 °C with urea, after 10, 20, 30 and 40 days of incubation. The values are the mean ± SE for each incubation period and are not significantly different (*P* ≤ 0.05) from each other according to Duncan’s multiple range (DMR) test

The results obtained from the present study stated that there was about 57–253 and 61–207 % increase in the urease activity in black soil observed over the control. This was noticed in the black soil after 10 days of incubation period, whereas in the case of red sandy loam soil, the corresponding figures of the percentage enhancement by the two selected fungicides, tebuconazole and copper hydroxide, at two levels were 63–203 and 43–156 % during the same period of incubation. However on comparison of tebuconazole and copper hydroxide at 5.0 kg ha^−1^, tebuconazole produced maximum stimulation in urease activity in black clay soil than red sandy loam soil in the present study. At higher concentrations, i.e., 7.5 and 10.0 kg ha^−1^, urease activity was significantly inhibited by treating the selected soil samples with both the selected fungicides. Among the two fungicide treatments, tebuconazole produced a different stimulation over control. In the present study, comparatively, the black soil showed higher enzyme activity than the red soil throughout the experiment. It can be concluded that the high enzymatic activities are associated with high organic matter content. Urease activity was significantly inhibited gradually at higher concentrations, i.e., 7.5 and 10.0 kg ha^−1^, in both the fungicide treatments with the incubation periods, i.e., 20, 30 and 40 days (Rangaswamy and Venkateswarlu 1996). In the present experiment, the maximum inhibition was recorded after 30 and 40 days of incubation periods. The inhibitory effect was reduced upon further incubation due to the reduction in the concentration and degradation of the applied fungicides. Ramudu et al. (2012) stated that the activity of urease, implicated in the hydrolysis of urea, was significantly enhanced under the impact of propiconazole and chlorothalonil up to 5.0 kg ha^−1^ level in both the soils. The two fungicides at 10, 25, 50 ppm levels individually cause 7–35 and 13–53 % increase in urease activity, respectively, compared to control in laterite soil and 28–54 and 12–52 %, respectively, compared to control in vertisol soil in a 10-day interval. For instance, Bending et al. (2007) reported that applications of azoxystrobin, tebuconazole and chlorothalonil affected the size and structure of microorganisms in the soils with low organic matter, but not in a comparable soil with high organic matter. The urease enzyme plays an important role in nitrogen cycling. Decrease in the enzyme activity results in the change in ion concentration, pH, and reduction of oxidation potential on solid organic and inorganic particles interspersed with mineral particles of larger size and inhibition of active site of urease enzyme by the selected fungicides. There have been reports on the effect of captan and trifloxystrobin on urease activity. The results obtained from the previous study suggest that the copper hydroxide alternatives, captan and trifloxystrobin, do not pose a short-term risk and are relatively more toxic than copper hydroxide to N cycling processes in soil. Similarly, urease activity was inhibited by napropamide at all concentrations relative to the control with long periods of pesticide application (Guo et al. 2008). In the same way, Cycon et al. (2010) noticed that urease activity declined in red sandy loamy soils with a combination (mancozeb + dimethomorph) at higher concentrations than control. Comparatively in the present study, the black soil showed higher urease activity than the red soil throughout the experiment due to its high organic matter content. Several studies reported that the stimulation of soil enzyme activities was associated with high organic matter content (Fig. 2).

## Conclusion

The results of the present study clearly indicated that the two selected fungicides, tebuconazole and copper hydroxide, profoundly enhanced both the phosphatase and urease activities at 1.0–5.0 kg ha^−1^. Based on the above results, it is concluded that the microbial activities (i.e., enzyme activities) were not affected by the fungicides applied at recommended levels in the agricultural system to control the pests.
